# Ocular abnormalities in Polish Hunting Dogs

**DOI:** 10.1371/journal.pone.0258636

**Published:** 2021-11-05

**Authors:** Ireneusz Balicki, Małgorzata Goleman, Agnieszka Balicka

**Affiliations:** 1 Department and Clinic of Animal Surgery, Faculty of Veterinary Medicine, University of Life Sciences in Lublin, Lublin, Poland; 2 Department of Animal Ethology and Wildlife Management, Faculty of Animal Sciences and Bioeconomy, University of Life Sciences in Lublin, Lublin, Poland; 3 Small Animals Clinic, University of Veterinary Medicine and Pharmacy in Košice, Košice, Slovakia; University of Miami, UNITED STATES

## Abstract

This study aimed to describe and determine the prevalence of ocular abnormalities in Polish Hunting Dogs. The study was conducted with 193 Polish Hunting Dogs: 101 female and 92 male animals, aged between 3 months and 12 years. Ophthalmic examinations were performed using slit lamp biomicroscopy, ophthalmoscopy, and tonometry based on the ophthalmological protocol for the examination of hereditary eye diseases. Spectral-domain optical coherence tomography (SD-OCT) was performed for dogs with sudden acquired retinal degeneration syndrome (SARDS) and progressive retinal atrophy (PRA), while electroretinography was also performed in dogs with SARDS. Five dogs (2.6%) were diagnosed with cataract, iris coloboma in 3 dogs (1.6%), ocular dermoid in 1 dog (0.5%), and retinal dysplasia, distichiasis and entropion in 1 dog (1%). Three dogs (1.6%) were diagnosed with PRA and SARDS occurred in 1 dog. Retinal lesions was observed in 16 dogs (8.3%). The clinical signs of retinopathy observed in Polish Hunting Dogs included discoloration of the tapetal fundus, patchy increased reflectivity in the region of discoloration, focus of hyperpigmentation and an area of tapetal hyper-reflectivity with a pigmented center. SD-OCT performed in the 3 dogs with PRA revealed alteration in the retinal layers, which was most advanced in the non-tapetal fundus. Although SD-OCT revealed retinal layers with normal architecture only in some parts of the dorsal, nasal and temporal regions in dogs with SARDS, areas of disorganized external limiting membrane, myeloid zone, ellipsoid zone, outer photoreceptor segment and interdigitation zone were also observed. Polish Hunting Dogs should undergo periodic ophthalmological examination for the evaluation of other hereditary eye diseases. The prevalence of retinal lesions in Polish Hunting Dogs requires further research.

## Introduction

The Polish Hunting Dog is one of five canine breeds indigenous to Poland that are recognized by the Fédération Cynologique Internationale **(**FCI). In October 1984, the Scientific Council of the Polish Kennel Club developed a preliminary breed standard based on zoometric measurements first performed in 1968 and repeated in 1982 [1, 2]. During the 2006 Euro Dog Show in Poznań, Poland this breed standard was acknowledged and assigned the status of “preliminarily approved”. The Polish Hunting Dog gained final approval on 7 November 2017, during a meeting of the FCI, which allowed dogs of the breed to compete for the title of international beauty champion’. The breed standard was registered under number 354 (FCI- 52 St. N° 354 / 23.11.2017).

Previous studies identified two progenitors of the Polish Hunting Dog breed, from whom a total of 1947 animals were descended. The breed was found to have 27 founders [3]. A study by Goleman et al. (2019) explored the genetic diversity of the Polish Hunting Dog population based on the microsatellite loci: the value of the polymorphic information content (PIC) was calculated to be 0.5552 with coefficient of inbreeding (FIS) at a population mean level of -0.012 [3]. The mean inbreeding for Polish Hunting Hounds was 0.1151, which was estimated using pedigree analyses. The value is relatively low, which suggests a correct selection of mating pairs and avoidance of inbreeding. The population mean kinship for the Polish Hunting Dog breed was 0.1198 [3]. Currently, Polish Hunting Dogs are not subject to any additional health-related breeding requirements (screening schemes, veterinary, or DNA) or field trials.

The development of veterinary medicine and ability to determine the carrier status for certain hereditary diseases have enabled the elimination of affected animals from the breeding cycle before they enter the reproductive stage. Therefore, it is necessary to identify diseases affecting the respective canine breeds, including ocular disorders. Detailed analyses focusing on the prevalence of ophthalmic diseases in various dog breeds have already been published [4, 5]. The Genetics Committee of the American College of Veterinary Ophthalmologists conducts an annual review of the scientific literature to identify statistical data related to ocular abnormalities in the respective dog breeds. The collected data are published in the “Blue Book: Ocular Disorders Presumed to Be Inherited in Purebred Dogs”, which is updated at intervals of several years (Genetics Committee of the American College of Veterinary Ophthalmologists) [4]. Unfortunately, the currently available studies are unable to provide data on dogs from indigenous Polish breeds, including Polish Hunting Dogs. At present, it is not mandatory to examine hereditary diseases in Polish breeds before introducing them into kennels.

Therefore, the aim of this study is to determine the prevalence and describe ocular abnormalities in Polish Hunting Dogs.

## Materials and methods

The study was conducted with 193 Polish Hunting Dogs: 101 females and 92 males, aged between 3 months to 12 years, weight (12-37kg). None of the dogs were neutered. The dogs were patients of the Department and Clinic of Animal Surgery at the University of Life Sciences in Lublin. All dogs included in the study were privately owned pet dogs, and the food given to the dogs was of different qualities and quantities based on the owners’ instructions. For all dogs water was given *ad libitum*. As patients of the Department and Clinic of Animal Surgery at the University of Life Sciences in Lublin, these Polish Hunting Dogs were examined as regular patients of the clinic, as well as due to recommendations from the kennel club, for ocular examination prior to breeding. Some dogs were included in the examination of ocular abnormalities in Polish hunting dogs. All dogs were healthy, under regular medical care, regularly vaccinated and dewormed according to the schedule. Examination or eventual veterinarian consultations were performed as necessary. All owners consented to the use information regarding the health status of their dogs in this study. The consent was obtained verbally. For additional ERG and SD-OCT examinations, consent was obtained in written form. Research was approved by the Scientific Research Committee of the Department and Clinic of Animal Surgery at the University of Life Sciences in Lublin (#4/2018) concerning non-experimental clinical patients. The study was performed in accordance with the Polish law and with Directive 2010/63/EU of the European Parliament and of the Council of 22 September 2010 on the protection of animals used for scientific purposes, Chapter I, Article 1, point 5(b).

### Ophthalmic examinations

Ophthalmic examinations were performed using slit lamp biomicroscopy (Shin Nippon; Ohira Co., Ltd, Niigata, Japan) and tonometry (Tonovet; Icecare, Finland). Ophthalmoscopy was conducted using a direct ophthalmoscope (Welch Allyn, NY, USA), indirect ophthalmoscope (Keeler; Windsor, UK), and panoptic ophthalmoscope (PanOptic; Welch Allyn, NY, USA), following pupillary dilatation with 1% tropicamide (Tropicamidum 1%; WZF Polfa S.A., Warsaw, Poland). The dogs were also subjected to a thorough clinical examination to eliminate the possibility of other diseases. All dog owners were comprehensively interviewed. Each dog was examined according to the ophthalmological protocol for the examination of hereditary eye diseases. Ocular fundus photography was performed using a fundus camera (Handy NM 2000; Nidek, Tokyo, Japan) connected to a computer with software (IrfanView) that permitted direct analysis of the examined areas and cataloguing of the results of particular exams. Ophthalmic examinations included testing for the menace response, dazzle reflex, visual placing, and visual tracking. Chromatic pupillary light reflexes (cPRL) were also examined using the BPI-50 Precision Illuminator (RetinoGraphics Inc, USA).

### Optical coherence tomography

Retinal examinations were conducted using spectral-domain optical coherence tomography (SD-OCT) (SD-OCT Topcon 2000, Topcon Corporation; Tokyo, Japan) in a male dog diagnosed with retinopathy and sudden acquired retinal degeneration syndrome (SARDS), and a female dog suffering from progressive retinal atrophy (PRA). An spectral-domain optical coherence tomography (SD-OCT) was performed on the day of SARDS diagnosis. The control group for the comparison of the SD-OCT results for the dog with SARDS comprised 9 dogs of various breeds aged between 11 and 12 years, while the control group comprised 8 dogs of various breeds aged between 7 and 8 years for the dog diagnosed with PRA.

The dogs were sedated with medetomidine 0.03 mg/kg (Cepetor 1 mg/mL, CP Pharma, Germany) administered intramuscularly. Local anaesthesia of the corneal surface was achieved using 0.4% proxymetacaine hydrochloride (Alcaine 5 mg/mL, Alcon). The pupils were dilated with tropicamide eye drops (Tropicamidum WZF 1%, Polfa Warszawa S.A.). The cornea was hydrated with a saline solution, which was applied every 20–30 s, during SD- OCT examination.

Radial and linear scans were performed for the morphological and morphometric evaluation of the retina. The scans were performed dorsal, ventral, nasal, and temporal to the optic disc. Dorsal and ventral linear scans were performed 5000–6000 μm from the optic disc, whereas nasal and temporal linear scans were performed 6000–7000 μm from the optic disc. The dorsal and ventral linear scans were performed parallel to the horizontal diameter of the optic disc. The temporal and nasal scans were performed at the upper middle-third of the vertical diameter of the optic disc. Retinal thickness was determined as the mean of the thickest and thinnest sections of the retina in the region with apparent localized retinal atrophy. The device’s software, Topcon 3D OCT 2000, facilitated determination of the exact site of measurement. Measurements of the following retinal layers were obtained manually using the SD-OCT software’s caliper function: internal limiting membrane (ILM) + nerve fiber layer (NFL) + ganglion cell layer (GCL), inner plexiform layer (IPL), inner nuclear layer (INL), outer plexiform layer (OPL), outer nuclear layer (ONL), photoreceptor layers (PRL) external limiting membrane (ELM) + myeloid zone (MZ) + ellipsoid zone (EZ) + outer photoreceptor segment (OPRS) + interdigitation zone (IZ), outer retina (OR) (OPL + ONL + ELM + MZ + EZ + OPRS + IZ), and total retinal thickness (TRT) (ILM to the IZ without the retinal pigment epithelium).

### Electroretinography

The dog with SARDS also underwent electroretinography (ERG). Electroretinography examination was performed 3 days after SD-OCT. The electroretinograms were recorded using a one-channel ERG unit (RETIportERG; Acrivet, Hennigsdorf, Germany) as described previously [6].

### Pedigree analysis

A pedigree analysis was performed for the 3 dogs diagnosed with PRA. The analysis was conducted using the Contribution, Inbreeding (F) and Coancestry 1.0 software in accordance with the relevant guidelines [7]. The pedigree analysis covered all the ancestral generations registered in the Polish Pedigree Book [*Polska Księga Rodowodowa* (PKR)] and Preliminary Book [*Księga Wstępna* (KW)] since the inclusion of the breed in 193.

## Results

**Fig 1:**
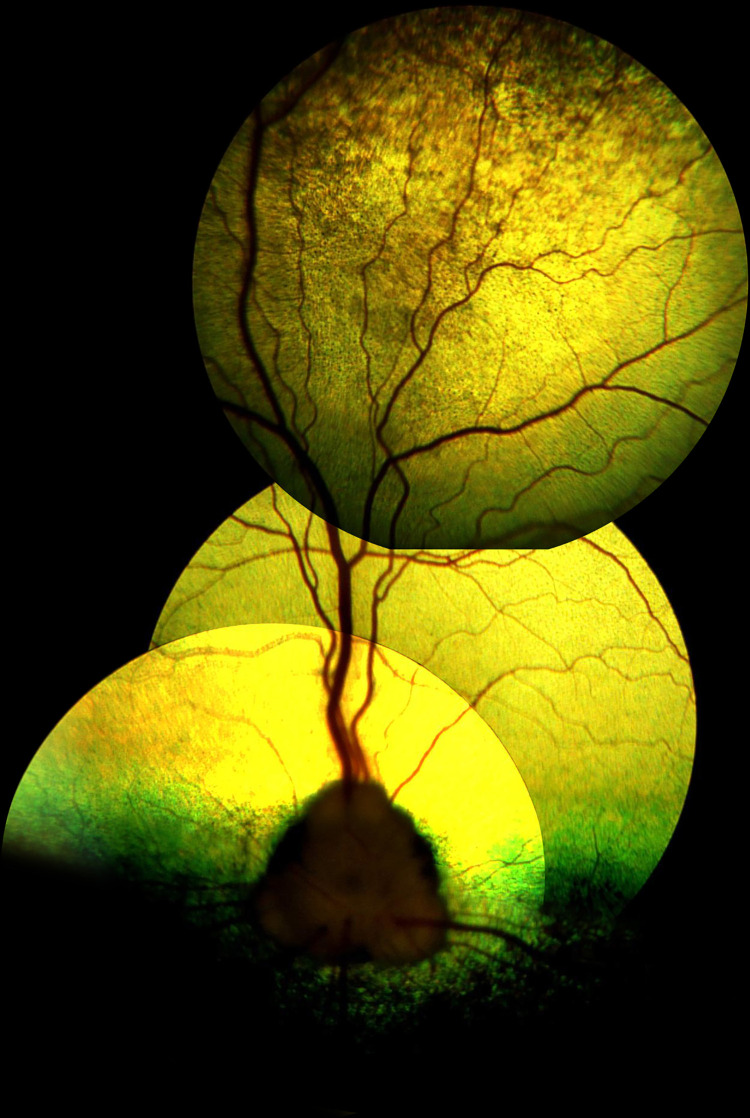
Discoloration of the tapetal fundus in dog number 3, diagnosed with retinal lesions.

**Fig 2:**
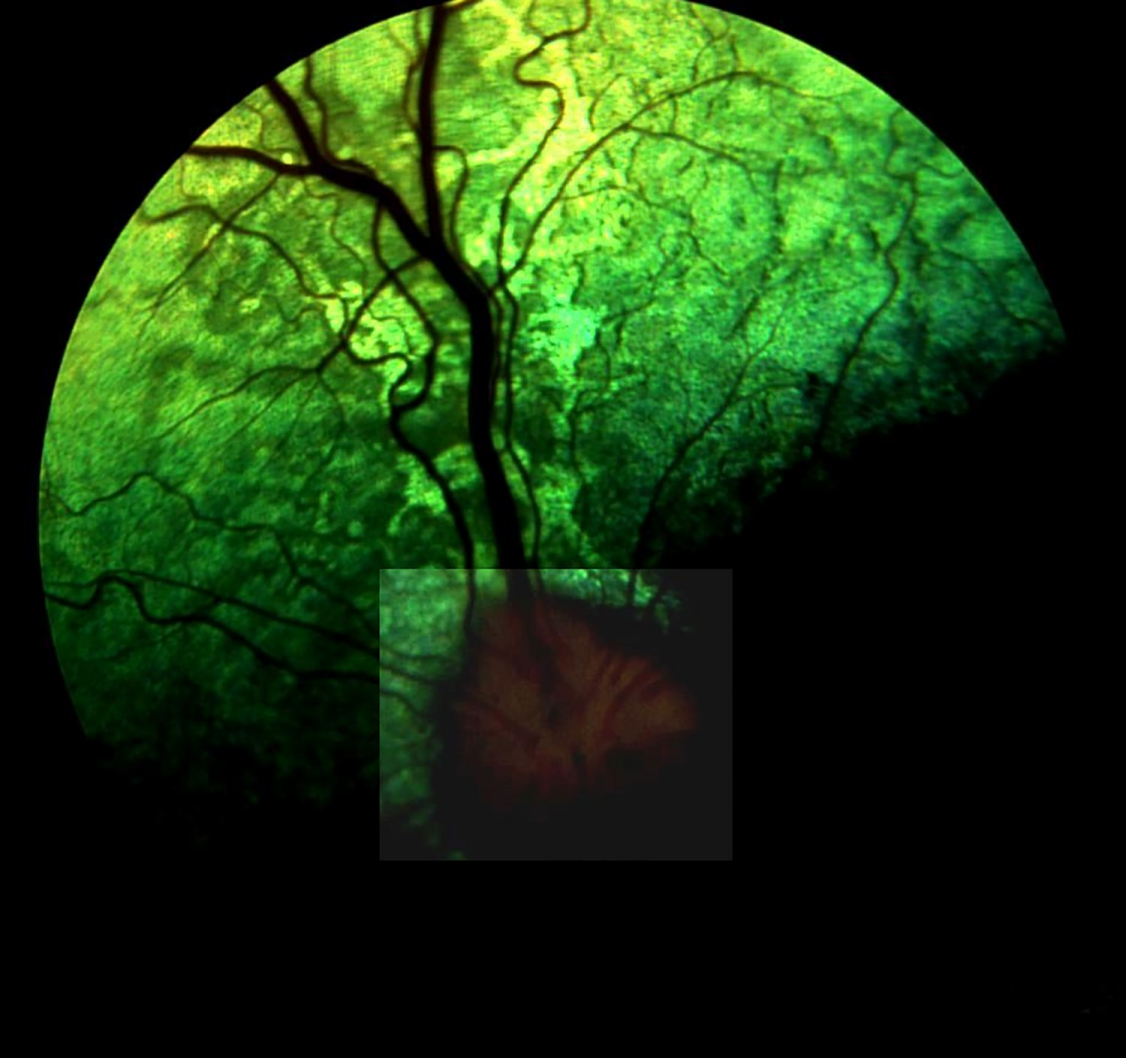
Discoloration of the tapetal fundus with irregular foci of hypereflection in dog number 9, diagnosed with retinal lesions.

**Fig 3:**
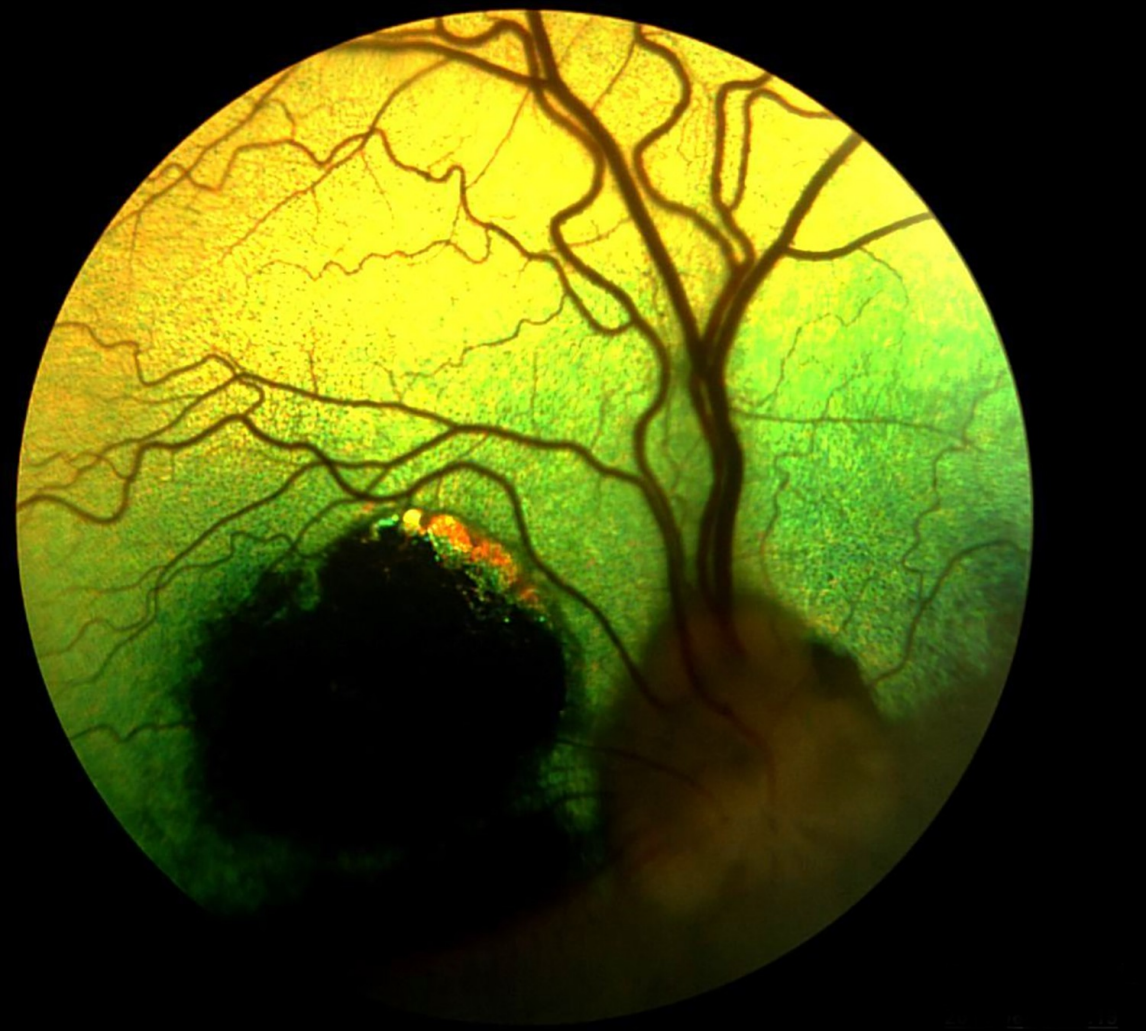
The hyperpigmentation with spreading halo of hyperreflection localized dorsally in dog number 10, diagnosed with retinal lesions.

**Fig 4:**
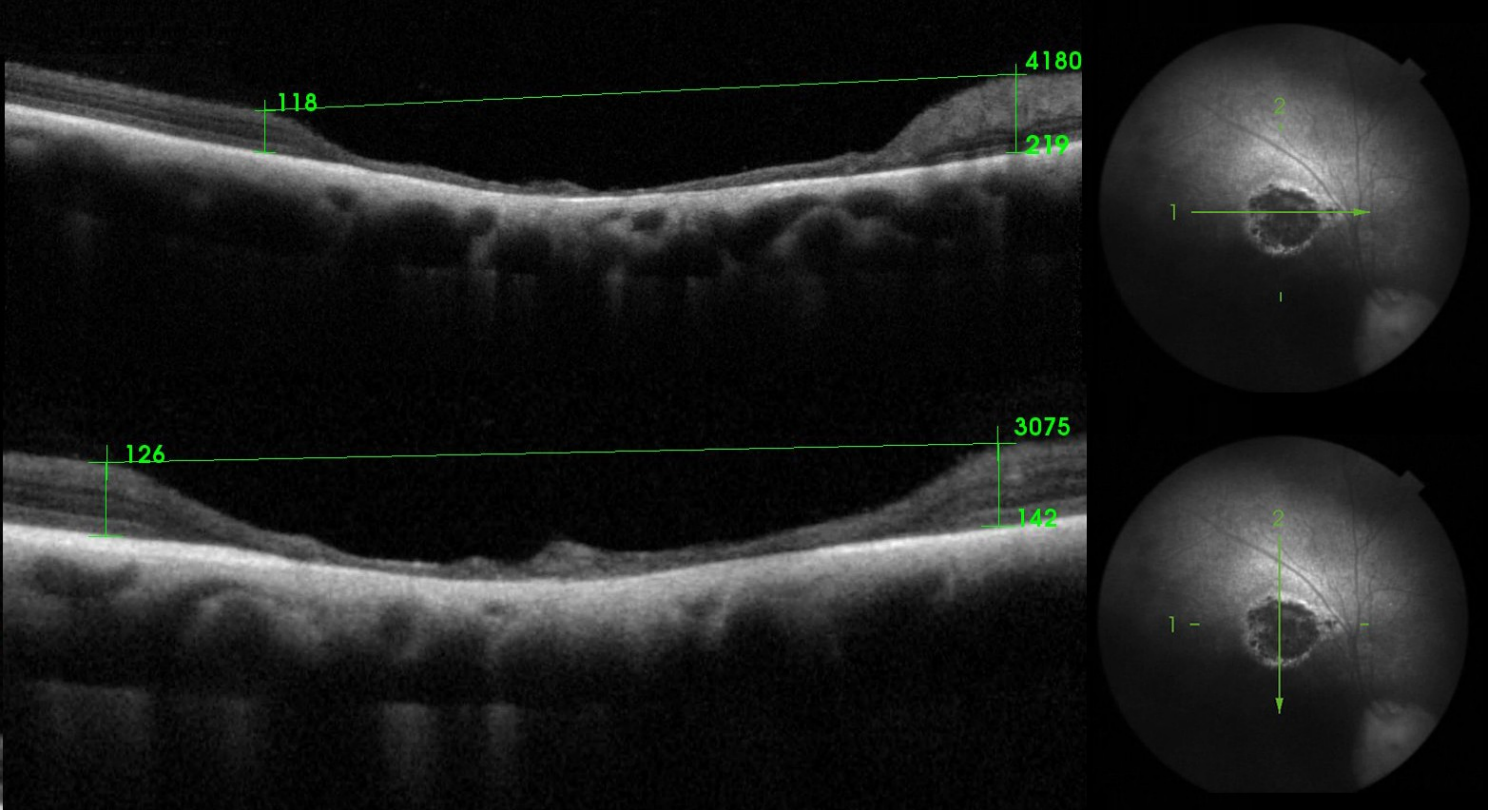
Measurement of size (length and width) of retinal lesion and its rim thickness (TRT), in dog number 16, also diagnosed with SARDs.

The ocular abnormalities identified in the Polish Hunting Dogs included in this study are presented in Table 1. Retinal lesions was the most commonly diagnosed ocular condition. Retinal lesions were observed in 16 dogs, i.e., 8.3% of the study population (8 cases of unilateral and 7 cases of bilateral retinal lesions) (Table 2). Retinal lesions were diagnosed in 10 male and 6 female dogs aged between 5 months and 10 years. The observed signs of retinal lesions in Polish Hunting Dogs included discoloration of the tapetal fundus, patchy increased reflectivity in the region of discoloration, and strongly pigmented foci with adjacent hyper-reflective foci (Figs 1–3). Dog number 16, who was diagnosed with SARDS, additionally suffered from unilateral retinal lesions characterized by an area of tapetal hyper- reflectivity with a pigmented center. Retinal SD-OCT revealed concomitant advanced atrophy of the respective retinal layers in the region that appeared as an area of tapetal hyper- reflectivity, with a pigmented center during ocular examination, in the animal diagnosed with SARDS. The retinal lesions measured 4180 μm in width and 3075 μm in height (Fig 4). The thickness of the retina at the dorsal rim, ventral rim, nasal rim, and temporal rim of retinal lesions was 126 μm, 142 μm, 219 μm and 118 μm, respectively. Cataracts were observed in 5 animals: 3 dogs aged 3, 4, and 8 years were diagnosed with immature bilateral nuclear and cortical cataract, and 2 dogs aged 9 and 10 years were diagnosed with mature bilateral nuclear and cortical cataract. Due to mature bilateral cataracts, impaired vision was observed in both dogs. In both dogs, the menace response was negative, as was tracking. Dazzle reflex, pupillary light reflexes and cPLR for red and blue light were present. The dogs had difficulty navigating an obstacle course test under scotopic and photopic conditions.

**Table 1:** Percentage of ocular abnormalities in examined Polish Hunting Dogs.

Ocular abnormalities	Number of animals	Percentage of examined dogs
Retinal lesion	16	8,3%
Cataract	5	2,6%
PRA	3	1,6%
Iris coloboma	3	1,6%
Distichiasis	2	1%
Retinal dysplasia	2	1%
Dermoid	1	0,5%
Entropion	1	0,5%
Eversion of nictitating membrane	1	0,5%
SARDS	1	0,5%

**Table 2:** Retinal lesions.

Dog no.	Age (years)	Sex	Fundus examination	
			OD	OS
1	2	**♂**	Discoloration of the tapetal fundus	
2	2	**♀**	Normal	Oval focus of hyperpigmentation with a smaller adjacent focus of hyperreflectivity, dorsally relative to the optic disc, the size of the optic disk
3	2	**♀**	Discoloration of the tapetal fundus	
4	2	**♀**	Discoloration of the tapetal fundus	
5	5 months	**♂**	Discoloration of the tapetal fundus	
6	9 months	**♀**	Discoloration of the tapetal fundus and foci of hyperpigmentation with irregular borders located in the vicinity of blood vessels	Normal
7	8	**♀**	Discoloration of the tapetal fundus also located along the blood vessels	Normal
8	10	**♂**	Discoloration of the tapetal fundus	
9	3	**♂**	Discoloration of the tapetal fundus with irregular hyperreflective foci	Normal
10	10	**♂**	Oval focus of hyperpigmentation with an area of hyperreflectivity in its upper part, temporally to the optic disc and 1 the size of the optic disc	Normal
11	7 (1^st^ exam) 9 (2^nd^ exam)	**♂**	1^st^ exam–normal 2^nd^ exam—discoloration of the tapetal fundus	1^st^ exam–normal 2^nd^ exam—discoloration of the tapetal fundus
12	2	**♀**	Oval focus of tapetal hyper- reflectivity with pigmented center located dorso-temporal to the optic disc, 1/25 the size of the optic disk	Normal
13	3	**♂**	Discoloration of the tapetal fundus	
14	1,5	**♂**	Normal	Discoloration of the tapetal fundus with foci of abnormal pigmentation along the blood vessels
15	3	**♂**	Normal	Discoloration of the tapetal fundus
16	12	**♂**	Moderate arteriolar attenuation and irregular constriction and dilatation of venules in the retina, area of the tapetal hyper-reflectivity with a pigmented center	Moderate arteriolar attenuation and irregular constriction and dilatation of venules in the retina.

**Fig 5:**
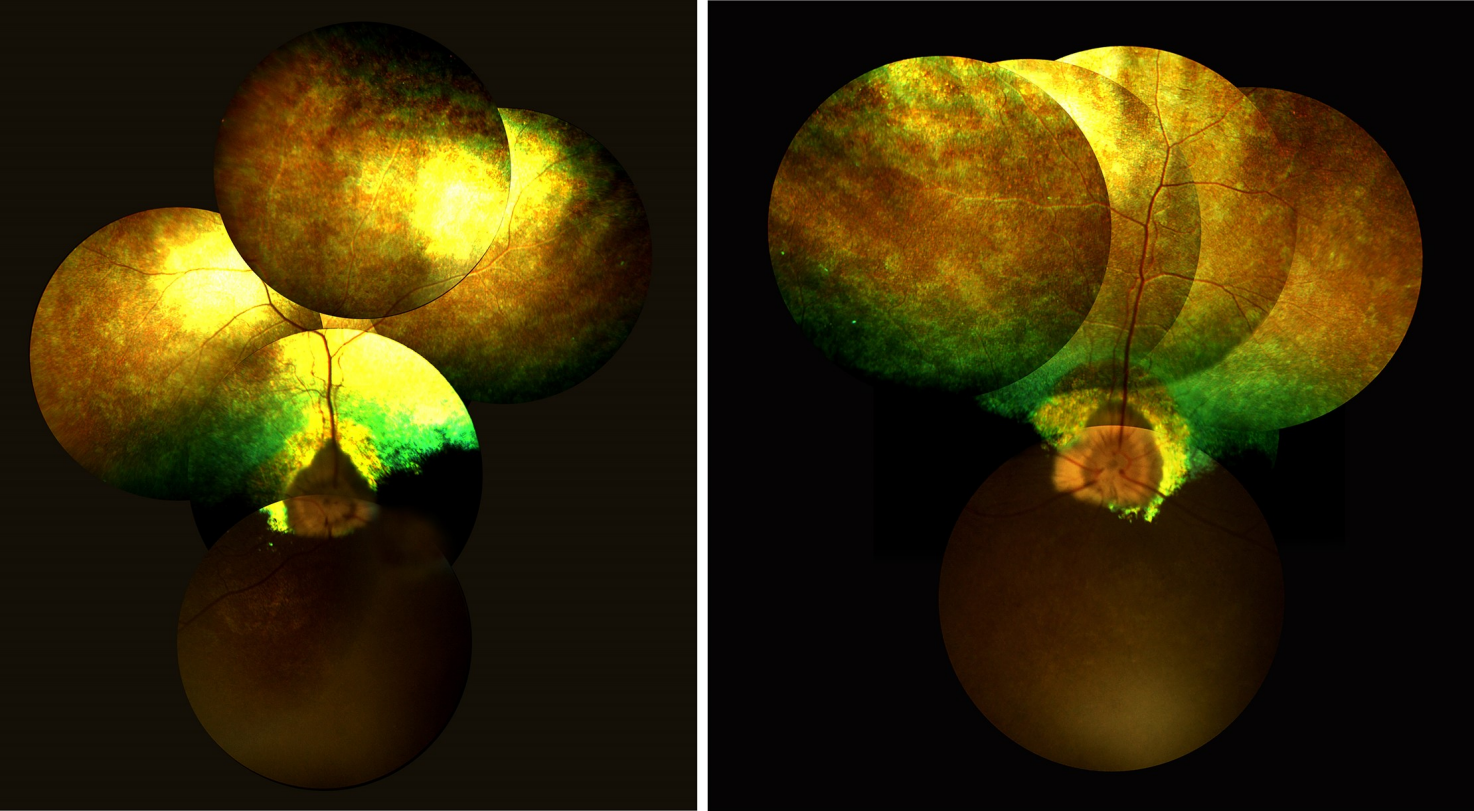
Symmetrical funduscopic changes in dog PRA 3. A- right eye, B- left eye.

**Fig 6:**
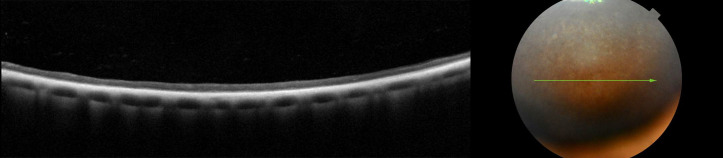
Advanced retinal atrophy presented as homogenous, thin hyyperrefectiv band on SD-OCT scan in dog PRA 3.

**Fig 7:**
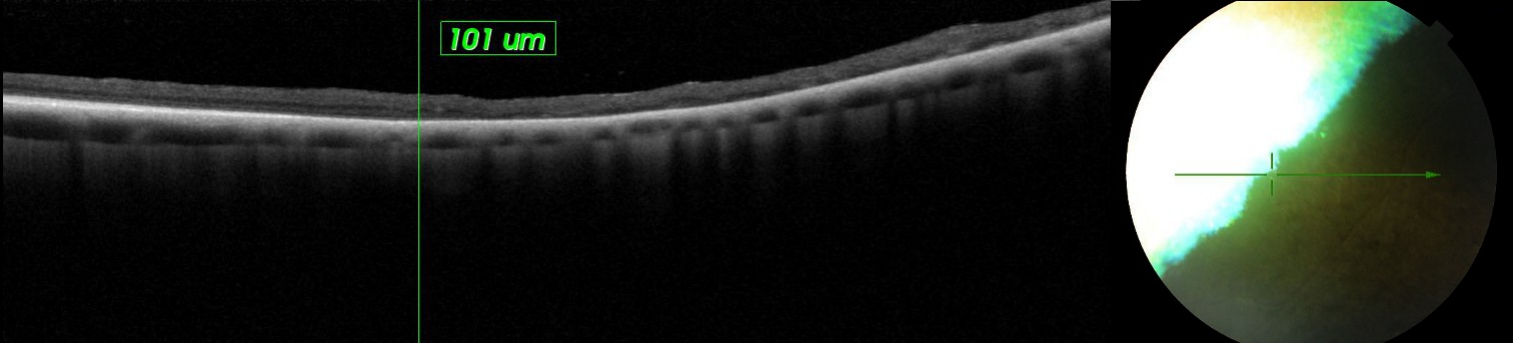
Disorganization of the retinal layers and atrophy pronounced in the nontapetal fundus, in dog PRA 3. Green vertical line on SD-OCT scan indicates localization on fundus.

**Fig 8:**
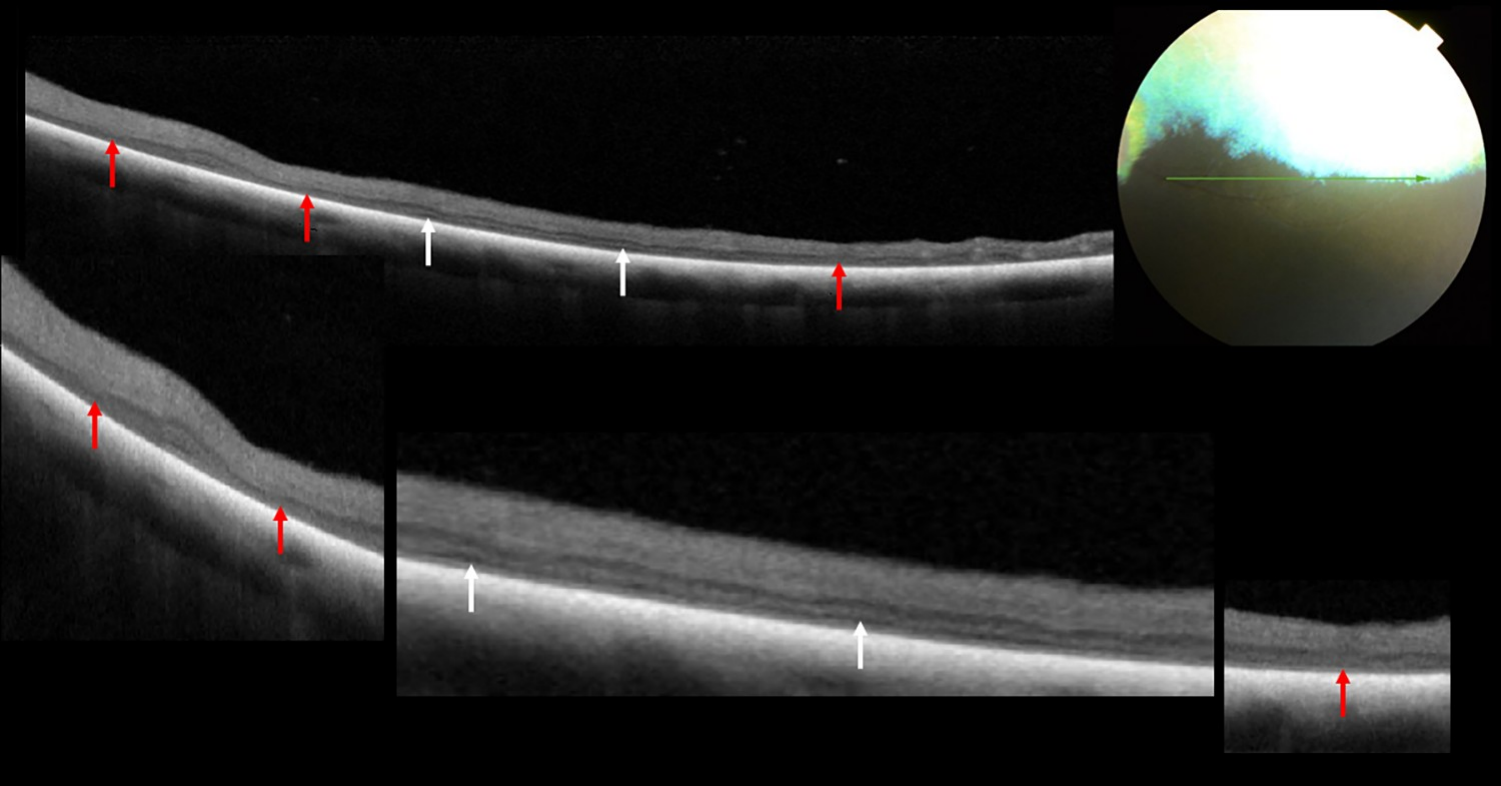
**Complete disorganization of outer retina and the blurring of their respective borders (red arrows).** The INL OPL and ONL layers are visible, the ELM, MZ, EZ, OPRS, and IZ bands have lost definition (white arrows) in dog PRA 3. Unilateral coloboma of the iris was diagnosed in 3 dogs aged 2, 6, and 7 years.

PRA was diagnosed in 2 male dogs aged 3.5 (PRA 1) and 6.5 (PRA 2), years as well as one female dog aged 7.5 years (PRA 3). No ophthalmic disorders were diagnosed during a previous examination, which was conducted when the female dog was 2 years old. All owners of the dogs diagnosed with PRA reported having observed vision impairment in the scotopic condition. The ophthalmic examinations in dogs PRA 1 and PRA 2, which included testing for the menace response, dazzle reflex, tracking, and visual placing, confirmed the absence of vision impairment. These dogs had difficulty navigating the obstacle course test under scotopic conditions but did not have difficulty in photopic conditions. Vision impairment was observed in the scotopic and photopic conditions in dog PRA 3. Chromatic pupillary light reflexes, which were examined with BPI-50, were present in dogs PRA 1 and PRA 2 under red and blue light, and were absent under red light, but present under blue light in dog PRA 3. The symptoms of PRA were identical in all the animals: tapetal hyperreflectivity, discoloration of the tapetal fundus, vascular attenuation, and irregular constriction and dilatation of venules in the retina (Fig 5). The symptoms occurred bilaterally and were identical in the left and right eyes. SD- OCT revealed disorganization of the retinal layers and significant TRT thinning in all regions of the fundus (Table 3) in the right and left eyes in dog PRA 3. The most advanced lesions were observed in the ventral region of the non-tapetal fundus, where it was impossible to distinguish between the respective retinal layers, and the retina appeared as a homogeneous, thin, hyperreflective band (Fig 6). Disorganization of the retinal layers was observed in the tapetal fundus, which appeared completely disorganized at the periphery of the non-tapetal fundus (Fig 7). Complete disorganization of outer retinal layers was observed in some region of fundus, which resulted in the blurring of their respective borders. The INL OPL and ONL layers remained visible, while the ELM, MZ, EZ, OPRS, and IZ bands appeared to have lost definition (Fig 8). The above-mentioned layers had merged into a single, hyper-reflective layer. Each of the dogs with PRA had had at least 2 litters. Dogs PRA 1 and PRA 3 were brothers from 2 subsequent litters from the same parents, each having fathered 4 subsequent litters (Table 4).

**Table 3:** Total retinal thickens measured on SC-OCT scans in control group and dog number 3 affected with PRA (μm).

TRT	Dorsal		Ventral		Temporal		Nasal	
	R	L	R	L	R	L	R	L
Control	163	165	141	149	172	172	158	160
PRA	114	106	54	57	104	107	104	110

**Table 4:** Pedigree analysis in three dogs diagnosed with PRA and they average relatedness with breed population.

Number of dog	Age in the day of ophtalmic examination (years)	Sex	Noumber of litters	Total number of pups	Average relatedness
PRA1	6,5	♂	4	21	0.185
PRA2	3	♂	4	22	0.100
PRA3	7,5	♀	2	9	0.120
Average relatedness of dogs suffering from PRA with the entire Polish Hunting Dog population 0.135					0.135

**Fig 9:**
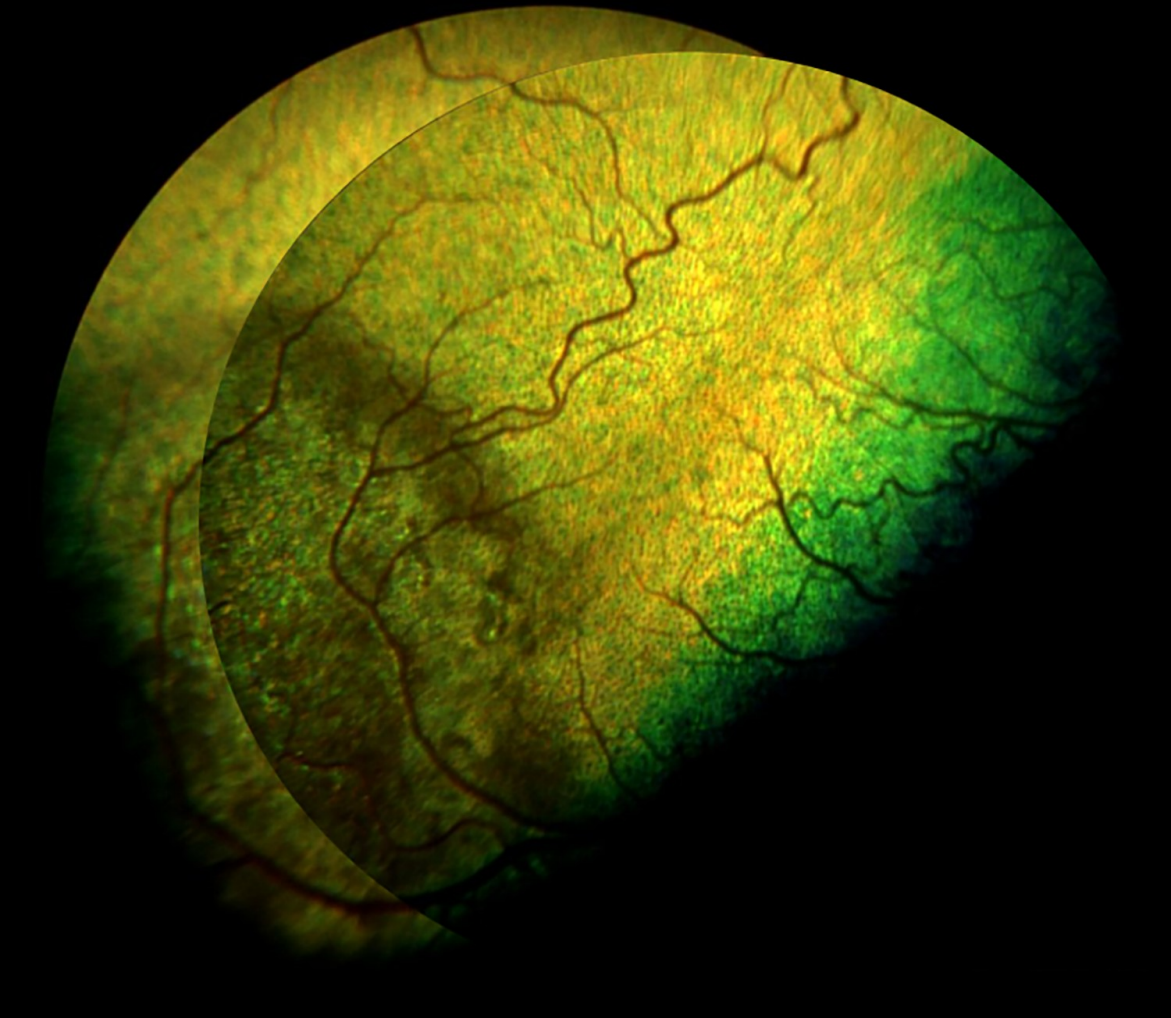
Geographic retinal dysplasia diagnosed in a 6-month-old female dog.

**Fig 10:**
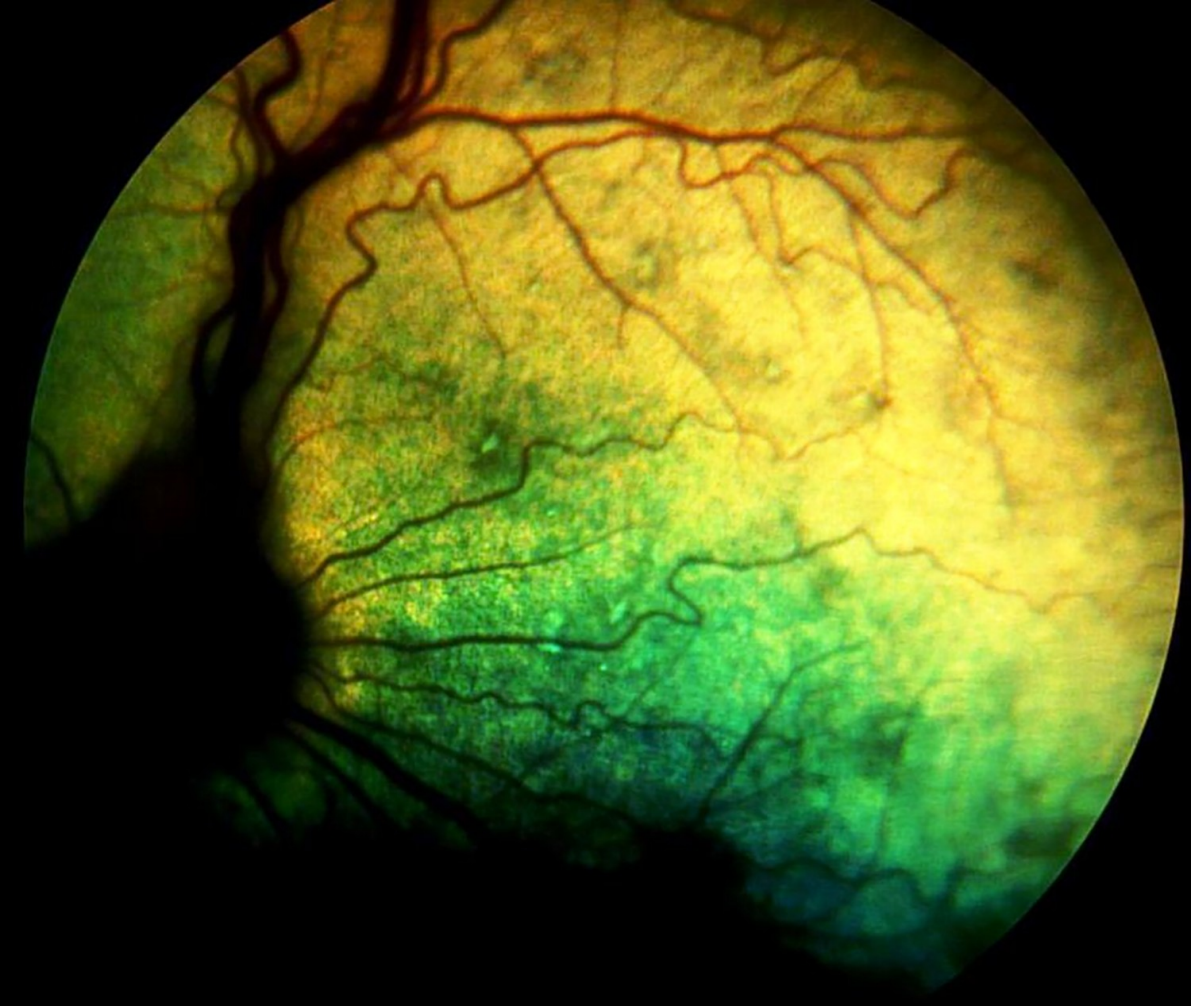
Multifocal retinal dysplasia with retinal folds diagnosed in a 1.5-year-old male dog.

Distichiasis was observed in the lower right and left eyelids in a 2-year-old male and in the right upper eyelid in a 4.5-year-old male dog. Unilateral retinal dysplasia was observed in 2 dogs. A 6- month-old female dog was diagnosed with geographic retinal dysplasia (Fig 9) while a 1.5- year-old male had multifocal retinal dysplasia with folds (Fig 10). A 3-year-old male dog was diagnosed with a corneal dermoid in the right eye, located in the lateral quadrant.

Bilateral entropion of the lateral canthus and medial region of the lower eyelid was diagnosed in a 1.5-year-old male dog. Unilateral eversion of nictitating membrane was diagnosed in an 8-month-old male dog.

**Fig 11:**
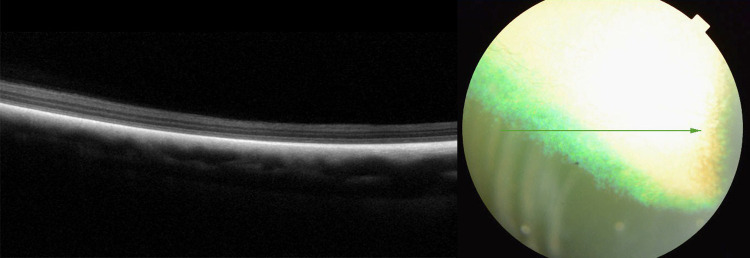
Retinal layers with visible architecture. Loss of definition of ELM band in dog diagnosed with SRADs.

**Fig 12:**
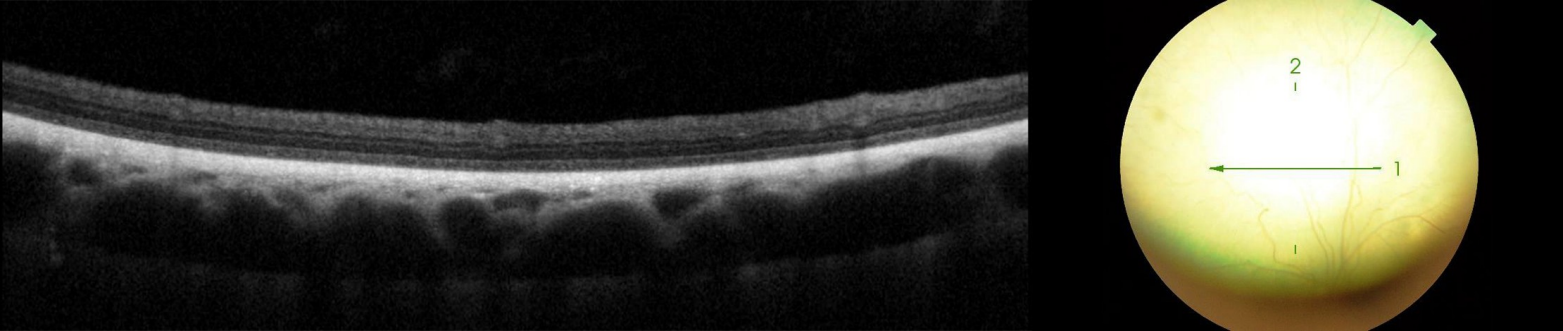
Loss of definition of ELM, MZ, EZ, OPRS and IZ band in the tapetal fundus, in dog diagnosed with SARDs.

SARDS was diagnosed in one 12-year-old male dog. Fundus examination revealed moderate arteriolar attenuation and irregular constriction and dilatation of venules in the retina. Chromatic pupillary light reflexes, which were examined using BPI-50, were absent under red light and present under blue light. Sudden loss of vision was observed. The dog with SARDS was blind and the a‐ and b‐waves were extinguished on ERG. Morphological evaluation of all the retinal regions using SD-OCT examination revealed that the most advanced lesions were located in the ventral region of the left and right eyes. Complete disorganization and atrophy of all retinal layers were observed. The retina was also the thinnest in these regions. Retinal layers with visible architecture could be observed only in some parts of the dorsal, nasal and temporal regions. The retinal layers were visible in these areas, but some of the OR layers were disorganized. Loss of definition of the ELM band was observed (Fig 11). The ELM, MZ, EZ, OPRS, and IZ appeared as a uniform hyperreflective layer in several regions of the tapetal fundus. The boundary between these layers was invisible (Fig 12). Morphometric evaluation revealed significant thinning of the ONL, PRL, OR, and TRT (Table 5).

**Table 5:** Retinal layer and total retina thickens measurements (μm) in dog affected with SARDS in which stage of disease allowed for measurement.

Retinal layers	Dog with SARDS								Control group							
	Right retina				Left retina				Right retina				Left retina			
	Dorsal	Ventral	Temporal	Nasal	Dorsal	Ventral	Temporal	Nasal	Dorsal	Ventral	Temporal	Nasal	Dorsal	Ventral	Temporal	Nasal
NFL+GCL	31		25	24	26		24	24	30	19	26	24	28	21	27	24
IPL	25		22	30	24		25	32	24	24	29	28	28	21	29	26
INL	13		10	12	10		11	14	10	9	12	11	11	10	12	11
OPL	10		11	12	10		10	10	11	10	11	11	11	11	11	11
ONL	21		18	19	17		17	20	42	32	41	38	43	30	44	38
PRL	30		31	31	29		30	30	39	37	42	39	39	36	41	39
OR	61		60	62	56		57	60	92	79	94	88	93	77	96	88
TRT	130	96	117	128	116	87	117	130	156	131	161	151	160	129	164	149

## Discussion

The unavailability of data on the prevalence of hereditary ocular abnormalities in Polish Hunting Dogs necessitated a study that included a sizable number of animals from this breed. It was not possible to correlate the results with the hereditary defect certificates issued to owners after ophthalmic examination, as such certified examinations were rarely conducted in this breed prior to this study. Therefore, research on the ocular abnormalities in Polish Hunting Dogs required considerable logistical and organizational preparation, because this study was conducted across various regions of Poland over the course of 4 years. This allowed us to include numerous dogs in the study sample: 193 animals of various ages (between 3 months and 12 years), which guaranteed a comprehensive review of the actual prevalence of ocular abnormalities in Polish Hunting Dogs. The investigation presents cross-sectional data. Overall, ocular abnormalities were diagnosed in 18.1% of the study population. The Bloodhound, and Black and Tan Coonhound breeds also present with ocular disorders that are similar to those in Polish Hunting Dogs. These breeds are susceptible to cataract and retinal dysplasia, akin to Polish Hunting Dogs. Both Bloodhounds, and Black and Tan Coonhounds suffer from retinal dysplasia with folds [4]. The retinal folds and geographic dysplasia and their corresponding clinical manifestations in Polish Hunting Dogs did not differ from the signs of retinal dysplasia in other breeds.

Retinal lesion was the most common type of ocular abnormality in this study population and was observed in 8.3% of the dogs. Acquired retinopathies often result from chorioretinitis and posterior uveitis. Inactive chorioretinitis is often characterized by areas of tapetal hyper- reflectivity with visible accumulation of black pigment at the center [8–11]. Pigment accumulation occurs due to pigment proliferation in the retinal pigment epithelium [9]. Pigment accumulation surrounded by a hyper-reflective area may represent scarring caused by chorioretinitis that occurred at an earlier stage [8–11]. Focus of hyperpigmentation with adjacent hyperreflectivity were identified in 4 dogs diagnosed with retinal lesions in this study. A study on German Shepherds demonstrated the development of lesions with indistinct borders and strongly pigmented centers in retinopathies, which were characterized by an area of tapetal hyper-reflectivity with a pigmented center [6]. Abnormal pigmentation was also observed in German Shepherds. Retinopathies occurred in working German Shepherds subjected to stress. Polish Hunting Dogs were also working dogs, who suffering from stress. The common retinal abnormalities observed in Polish Hunting Dogs included discoloration of the tapetal fundus, with a varying degree of advancement. It was identified in the upper dorsal region of the fundus and along the blood vessels (dog number 7). It was present in the upper dorsal region of the fundus in 6 dogs, and in the entire tapetal fundus in 4 dogs. The first examination indicated no lesions in the fundus of the eye in dog number 11, whereas the second examination revealed discoloration of the tapetal fundus. This suggests that the discoloration developed over the course of 2 years in the case of this particular animal. It is noteworthy that discoloration located in the upper dorsal region of the tapetal fundus was diagnosed in a 5-month-old male dog (dog number 5). We hypothesize that discoloration can occur in the upper dorsal region of the fundus and progress towards the ventral region.

PRA manifests with typical clinical symptoms in Polish Hunting Dogs. SD-OCT facilitated differentiation between the degree of disease progression in the respective regions. The most advanced retinal degeneration was observed in the ventral regions. This corroborates the results of earlier studies that reported that layer disorganization and retinal atrophy were more advanced in the ventral region compared to the other regions of the retina in the case of PRA [12]. Histopathological examination revealed that photoreceptor atrophy was accompanied by apoptosis of the other retinal layers, i.e., the ONL and OPL, and eventually the INL and IPL in PRA [13]. SD-OCT examination indicated disorganization of all retinal layers in Polish Hunting Dogs suffering from PRA. The INL, OPL and ONL were only visible in the dorsal regions and were accompanied by the loss of definition of the outer retina. These findings suggest that changes occurred in the outer retina in Polish Hunting Dogs with advanced PRA.

The inheritance of progressive retinal atrophy depends on the breed of the dog [9, 14–16]. The nature of GRPA inheritance remains unknown in Polish Hunting Dogs, and there is no dedicated genetic test for this breed. Hence, PRA in dogs from this breed can only be diagnosed on the basis of clinical examinations and it is impossible to identify carriers. Pedigree analysis revealed that dogs suffering from PRA showed an average relatedness of 0.135 with the population, which is slightly higher compared to the mean relatedness for the entire Polish Hunting Dog population (0.1198), also calculated using pedigree analysis [3]. The issue of excessive relatedness may occur in certain kennel lines and may result in the emergence of genetic disorders. This is particularly important in the case of small populations susceptible to the effect of the so-called genetic bottleneck, which leads to reduced genetic diversity and increased incidence of adverse alleles, and consequently hereditary diseases such as PRA. SARDS affects middle-aged to elderly and often moderately overweight dogs, aged between 7–10 years [17–20]. In our study, SARDS was diagnosed in a 12-year-old male dog. Microscopic studies conducted by Acland et al. demonstrated a total absence of the outer photoreceptor segment in SARDS-affected retinas [17, 18]. The ONL was thin. SD‐OCT examinations indicated that dogs with SARDS had significantly thicker inner retinas, thinner ONL, and thicker inner/outer photoreceptor segments [21]. Other studies that entailed SD- OCT examination of the retina found loss of definition of all of the photoreceptor bands in cases with SARDS [22]. The TRT decreased significantly in the Polish Hunting Dog with SARDS. Our own research demonstrated significant thinning of the OR in all examined retinal regions of the right and left eyes. Layers of the inner retina were visible on the SD- OCT scans in the dorsal region, while the photoreceptor layers underwent disorganization. Examination of the pathological changes using light and electron microscopy revealed disorganized lamellae, absence of the outer segments, and amorphous material in the interphotoreceptor matrix [18, 23]. It can be assumed that these pathological changes were the cause of disorganization of the photoreceptor layers observed on the SD-OCT scans in the Polish Hunting Dog with SARDS.

The determination and publication of these results demonstrating the prevalence of ocular abnormalities in Polish Hunting Dogs will enable responsible breeders to make more informed decisions and reduce the prevalence of hereditary diseases in dogs of this breed.

## Conclusions

Polish Hunting Dogs should undergo periodic ophthalmological examinations to determine the presence of other hereditary eye diseases. The prevalence of retinal lesions in Polish Hunting Dogs requires further research.
